# High-risk area for migraine attacks — a new concept in migraine pathophysiology

**DOI:** 10.3389/fneur.2025.1569361

**Published:** 2025-04-07

**Authors:** Marianna Gabriella Rispoli, Maria Vittoria De Angelis, Donato Melchionda, Gabriele Manente

**Affiliations:** ^1^Neurology and Stroke Unit, “G. Mazzini” Hospital, Teramo, Italy; ^2^Neurology and Stroke Unit, “Santo Spirito” Hospital, Pescara, Italy; ^3^Neurology Unit, University of Foggia, Foggia, Italy

**Keywords:** migraine pathophysiology, homeostasis, hypothalamic dysfunction, antidromic stimulation, trigeminovascular system, high-risk area

## Abstract

Migraine is a common primary and often disabling neurological disorder, whose pathophysiology is still debated. It does not appear to be an isolated event of head pain but the consequence of recurrent disruption of healthy homeostasis in some brain functions. We propose a new theoretical model, focused on the existence of a “high-risk area” for migraine attacks, which can represent a potential target of non-pharmacologic treatment and prevention. We suggest that migraine arises from the combined effects of three primary factors, namely depressive or unstable mood, unrestful sleep and sympathetic-parasympathetic imbalance with parasympathetic prevalence, alongside with their temporal variability, potentially through dysfunction of homeostatic hypothalamic networks in susceptible individuals. Moreover, these three primary factors contribute to a state of low brain energy, that contains the high-risk area and represents the condition in which migraine attacks rise up. Wearable devices, self-administered questionnaires and clinical tools (i.e., polysomnography, pupillary light reflex, plasma catecholamines dosage) may be used to monitor autonomic nervous system function, mood and sleep and demonstrate the existence of the high-risk area. This will be helpful for patients to understand when they are about to enter in the high-risk area, in order to implement strategies to prevent migraine attacks. This approach would provide a significant advantage in terms of prevention and early treatment.

## Introduction

Migraine is a common primary and often disabling neurological disorder, experienced by approximately 10% of the population (1, 2). According to the International Headache Society (IHS), migraine attacks are recurrent and last 4–72 h. Typical characteristics of migraine attacks include unilateral location, pulsating quality, moderate or severe pain intensity, aggravation by habitual physical activity and an association with digestive symptoms (e.g., nausea, vomiting) and/or photo- and phonophobia (3). Migraine attacks can vary in intensity of pain and patterns of associated symptoms, demonstrating a broad variability in the clinical presentation (4). The classic migraine attack consists of three phases: the premonitory phase, the headache phase, and the postdrome (5). The premonitory phase may occur hours to days before an attack and is characterized by fatigue, irritability, concentration impairment, neck stiffness, sensitivity to light and noise, nausea, food cravings, difficulties in speaking and reading, mood disturbances and yawning (6). According to premonitory symptoms, some patients can correctly predict migraine attacks (7). Patients may experience postdromes, which include fatigue, concentration impairment, neck stiffness, and sensibility to light and noise, for days after the headache. Up to one-third of migraine patients suffer migraine aura, i.e., fully reversible focal visual, sensory, language and/or brainstem symptoms that gradually spread over 5 to 60 min and then disappear (8). The aura phase commonly precedes or accompanies headache (9). Even if the description of migraine attacks as a succession of stages over time is useful from a didactic point of view, the phases of an attack can be overlapping and variable (9).

A wide range of acute medications are now available for migraine treatment. However, they are mainly aimed at resolving pain, which is just one component, even if the most bothersome, of migraine attacks. Moreover, many patients have a lot of side effects, and the severity of their headaches does not improve significantly (10).

### Theories on migraine pathophysiology

The pathophysiology of migraine is still debated (Table 1) and has yet to be fully elucidated (11). It was first considered a vascular disorder originating from dilated meningeal vessels, due to characteristic throbbing pain (12, 13). However, there is conflicting evidence about how intracranial and extracranial vessels are ictally and interictally affected in migraine patients (14, 15). Likewise, migraine provocation studies have provided contrasting results on the role of vasodilators in the generation of migraine attacks (16–19). For example, vasoactive intestinal polypeptide (VIP) demonstrated migraine-inducing properties only after prolonged infusion (19). Subsequently, a series of laboratory experiments have suggested that migraine pain may be due to a sterile neurogenically driven inflammation of the dura mater (20). Therefore, the first branch of the trigeminal nerve, connecting peripheral nociceptors in the meninges to their terminations in the brainstem, was hypothesized as responsible for migraine attacks in susceptible individuals (2), according to the trigemino-vascular theory (21). Indeed, in experimental models, a mechanical, electrical or chemical activation of afferent C-fibers meningeal nociceptors *in vivo* leads to the release of vasoactive proinflammatory peptides, such as calcitonin gene-related peptide (CGRP) and substance P, which produce a vasodilation of meningeal blood vessels, plasma extravasation, and local activation of dural mast cells, with consequent neurogenic inflammation (21–23). CGRP emerged as a crucial mediator of migraine attacks, and an important therapeutic target. The infusion of CGRP triggers delayed migraine attacks in susceptible individuals (24). However, contradictory results have been reported regarding interictal CGRP levels: some studies have identified elevated CGRP levels in the peripheral blood of both episodic (25, 26) and chronic migraine patients (27) compared to healthy controls, while other studies have found no differences in serum CGRP levels between migraine patients and healthy individuals (28, 29). These discrepancies may partly be attributed to methodological factors (30). Since some migraine patients do not respond to treatments that either target CGRP or its receptor, other substrates have been explored for their potential implication in migraine pathogenesis: among them, other neuropeptides (i.e., pituitary adenylate cyclase-activating polypeptide, VIP, amylin, and adrenomedullin), nitric oxide, and phosphodiesterase-3 and -5 appeared able to induce migraine-like attacks through vasodilation of intracranial arteries in human provocation studies (31).

**Table 1:** Theories about migraine pathophysiology over time.

Theories of migraine pathophysiology	Caveats	References
*Vascular* – Migraine attacks result from platelet-derived serotonin, which decreases cerebral blood flow through vasoconstrictive properties. After the reuptake or metabolization of serotonin, the constricted blood vessels dilate, inducing the characteristic migraine pain.	Evaluations of arterial dilatation and cerebral blood flow during migraine attacks in humans have found no intra- or extracranial arterial dilatation or increase in cerebral blood flow. Not all vasodilators cause migraine.	(12, 14–17)
*Trigemino-vascular* – The trigeminal nerve is thought to be the primary origin of migraine headaches. Stimulation of the trigeminal nerve causes vascular dilatation through plasma protein extravasation (PPE). Neurogenic inflammation in the periphery was proposed to be the generator of migraine pain.	Blockade of neurogenic PPE is not predictive of antimigraine efficacy in humans, as evidenced by the failure in clinical trials of PPE blockers. Direct evidence for a dural inflammatory component in migraine is lacking.	(21, 22, 33–35)
*Neural* – Spreading depression (SD), a wave of complete neuronal and glial depolarization, is the electrophysiological substrate of migraine aura and triggers the headache through activation of trigeminal nerve afferents.	It is unclear how cortical SD is triggered in migraine patients without an underlying brain injury. Premonitory symptoms and signs are unexplained by this theory; however, they may result from SD in subcortical structures, such as hypothalamus, hippocampus, and amygdala.	(38, 40, 41)
*Central* – Migraine is conceptualized as a disorder of sensory network gain and plasticity, starting at the hypothalamus and trigeminocervical complex through antidromic firing. Changes in thalamic and thalamo-cortical activity, as well as widespread alterations in brain connectivity, may be crucial in the aberrant sensory processing underlying migraine attacks.	The mechanism(s) through which the hypothalamus may become ‘overactive’ and contribute to the sensitization of trigeminal nociceptors through antidromic stimulation in migraine remains unclear. Neuroimaging studies have sometimes yielded inconclusive results, failing to identify alterations specific to migraine.	(9, 44, 49, 54, 55)

The classical view suggests that nociceptive signaling involves peripheral generation and orthodromic propagation of the spikes to the brain centers (32). Although trigeminal activation with subsequent neurogenic inflammation continues to be discussed (33), there is no direct evidence supporting a peripheral inflammatory component in migraine. Therefore, the possibility of the functionally opposite, antidromic signal conduction in the meningeal system was previously considered (34, 35). There is bidirectional nociceptive traffic in meningeal afferents implicated in the generation of migraine pain: beyond orthodromic propagation, a spontaneous antidromic activity of the trigeminal nerve in meninges, essentially originating from the trigeminal ganglion, has been demonstrated (36). Similarly to orthodromic activation, antidromic signal conduction leads to the release of CGRP from C-fibers, dilation of meningeal vessels and degranulation of local mast cells (34), activating Aδ-fibers and, in turn, trigeminal ganglions, the trigeminocervical complex, and axonal projections ascending to the midbrain, thalamic and hypothalamic nuclei. Thus, the focus was set on the cortical wave of spreading depolarization, which causes the aura phase and releases molecules affecting neurons, glial cells, and blood vessels (37). These mediators may diffuse to the overlying leptomeninges, activate the trigemino-vascular system, and lead to the typical head pain of migraine attacks (38). Infections like COVID-19 have been suggested as possible modifiers of brain bioelectrical activity through still unknown mechanisms: preliminary observations suggest that they may be responsible for cortical spreading depression (CSD) and subsequent appearance of visual aura by increasing neuronal activity, especially in the occipital lobe (39). Other possible internal and external factors of CSD include hormonal changes in women, changes of day-night rhythm, strong sensory stimuli, hunger, stress or intense physical activity (40). Although CSD has been demonstrated to cause migraine aura and headache in the animal model, the evidence of a causal link between these entities is still lacking in humans (41). Moreover, most migraine attacks do not include the aura phase and migraine aura can occur without headache, indicating that aura is neither necessary nor sufficient for headache onset. Therefore, CSD may be absent or “silent” during most migraine attacks. “Silent” CSD may result from the depolarization of a non-eloquent cortex, or produce symptoms not typically classified as aura (42). Spreading depression in brain regions different from the cortex or disruption of normal thalamocortical oscillations by CSD have been suggested as an alternative model to canonical CSD (43).

Nowadays, migraine is conceptualized as a disorder of sensory network gain and plasticity, starting at the hypothalamus and trigeminocervical complex (2, 44). Since migraine may display a diurnal periodicity, be triggered by altered homeostasis and include yawning, food cravings and fatigue among prodromal symptoms, the hypothalamus has been investigated as a potential generator of migraine attacks (38, 45). According to PET and functional MRI studies of triggered and spontaneous migraine attacks, migraine patients show stronger functional connections between the hypothalamus and brain areas regulating sympathetic and parasympathetic functions in the hours preceding a headache attack (46, 47), explaining some of the hypothalamic-mediated autonomic symptoms accompanying migraine, such as facial flushing, lacrimation and nasal congestion (48). The mechanism(s) through which the hypothalamus may become ‘overactive’ and contribute to the sensitization of trigeminal nociceptors in migraine remains unclear (49). More complex midbrain/brainstem networks might contribute to the generation of migraine attacks. Physiological changes like stress, sleep deprivation and hypoglycaemia may lead to synaptic and network hyperactivity in brainstem-cortical and thalamo-cortical networks of susceptible individuals, altering cortical excitability via glutamate increase (23, 50–53). Indeed, electrophysiological studies have shown changes in the function of thalamo-cortical circuits during the premonitory phase (50). In addition, structural and functional imaging studies have revealed differences in thalamic and thalamo-cortical activity in migraine patients versus controls, both during migraine attacks and the interictal phase (52, 53). Therefore, changes in thalamic and thalamo-cortical activity may be crucial in the aberrant sensory processing underlying migraine attacks (9). Furthermore, resting-state functional MRI studies during interictal phase revealed widespread alterations in brain connectivity of migraine patients, consistent with changes in the function of multiple overlapping sensory and pain-processing circuits involving the cortex, thalamus, hypothalamus, brainstem, amygdala and cerebellum (9). However, neuroimaging studies have sometimes yielded inconclusive results (54), failing to identify alterations specific to migraine (55), highlighting the need for further research on the topic.

### Approaching migraine treatment: going beyond pain management

Two key aspects of migraine management should be carefully considered: (1) early intervention is more effective; (2) there is a significant need for alternative, non-pharmacologic approaches for both acute and preventive treatments (56). A recent observational study suggested that incident-morning migraine attacks may result from poorer sleep quality and decreased energy during the prior day. On the contrary, increased energy and greater average stress may lead to headache onset later in the day. Despite different patterns of predictors related to morning and later-day incident headaches, circadian rhythms seem to play a major role in the manifestations of headache (57). Since the most common triggers of migraine are stress, fasting, atmospheric changes, sleep-related factors, hormonal and mood fluctuations (58, 59), non-pharmacologic treatment strategies (i.e., setting routines, relaxation therapies, aerobic exercise, hydration, avoidance of fasting and stressful situations) have proven effective in reducing migraine burden or days (60). Recently, situational prevention—treating patients during the interictal phase before symptoms develop, in situations of increased risk for migraine attacks—is becoming more widespread, as the small molecule CGRP antagonists (gepants) are indicated for both the acute and preventive treatment of migraine (61). However, this approach exposes asymptomatic patients to medication and potentially to unnecessary risks. Moreover, there is no clear definition of periods of increased risk to date (62). Lastly, treatment strategies will only be effective if they focus on correcting the complex pathophysiological alterations underlying the disease before its onset.

### The “high-risk” area: a new reading of migraine attacks

**Figure 1:**
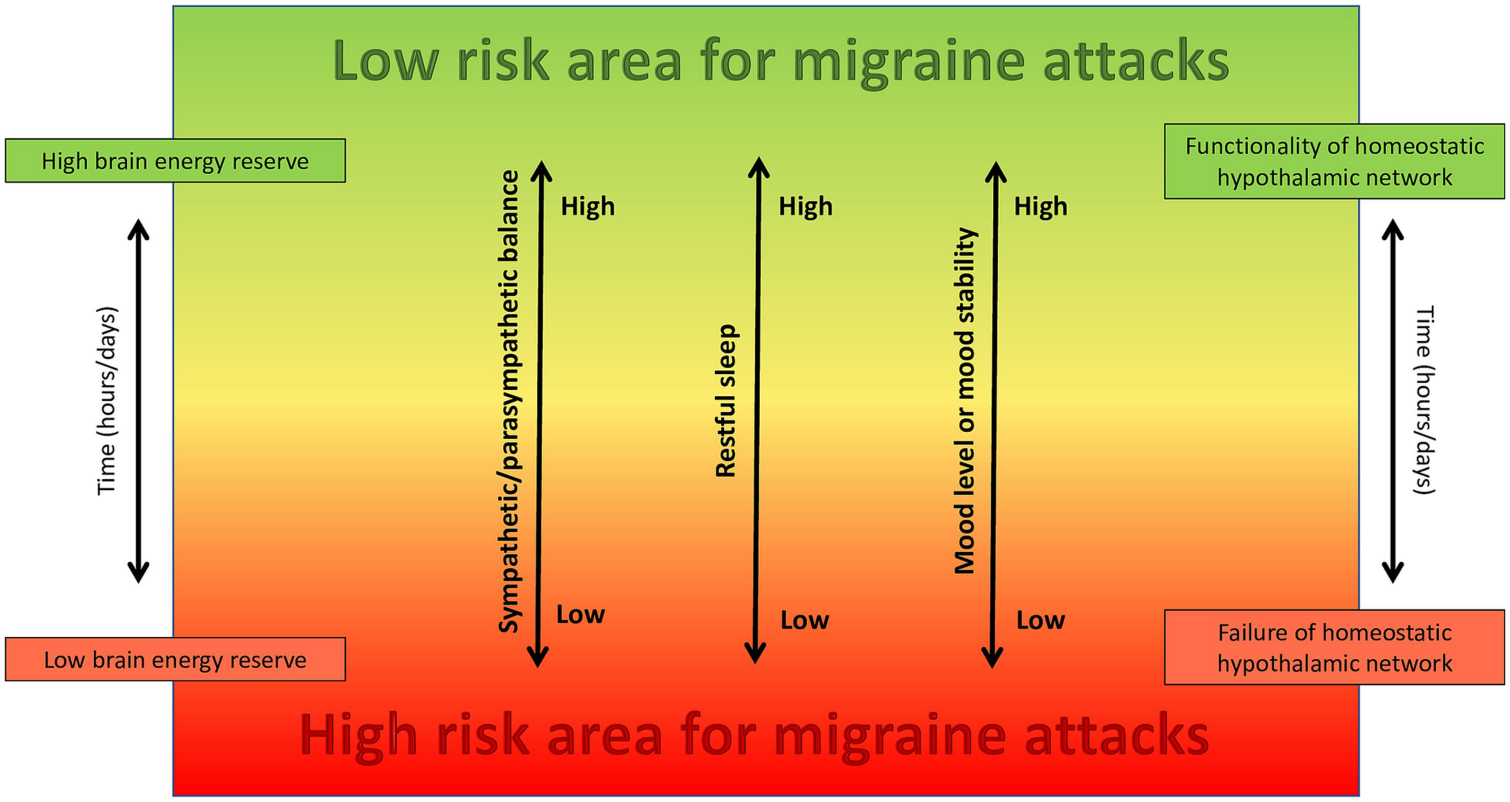
The combined effect of sympathetic/parasympathetic balance, restful sleep, and mood tone and stability, which are all regulated by the hypothalamus and associated neural networks, play a crucial role in determining brain energy levels and the likelihood of reaching the high-risk area for developing migraine attacks in susceptible individuals.

On this matter, we propose a new theoretical model focused on the existence of a “high-risk area” for migraine attacks onset, which can represent a potential target of non-pharmacologic treatment and prevention. We suggest that migraine arises from the combined effects of three primary factors, namely depressive or unstable mood, unrestful sleep and sympathetic-parasympathetic imbalance with parasympathetic prevalence, alongside with their temporal variability, potentially through dysfunction of homeostatic hypothalamic networks in susceptible individuals. Moreover, these three primary factors contribute to a state of low brain energy, that contains the high-risk area and represents the condition in which migraine attacks rise up (Figure 1).

It was suggested that migraine happens when the brain workload exceeds its energy reserve (63, 64). Indeed, the connectivity between hypothalamus and limbic areas, processing internal and external information, gradually increases toward migraine attacks (65). At some point, the hypothalamus loses its control over limbic structures, and this leads to a sensory overload of the deep brain regions (65). We hypothesize that this sensory overload could produce a spreading depression-like phenomenon, which involves non-cortical brain structures and activates migraine pain through antidromic pathways. This phenomenon may also be influenced by the activation state of some brainstem nuclei that modulate trigeminal nociceptive inputs (22, 45). For example, according to a diffusion tensor imaging study, the brainstem displays microstructural alterations throughout the migraine cycle. In particular, immediately before a migraine attack, mean diffusivity (MD) decreased in the spinal trigeminal nucleus, dorsomedial/dorsolateral pons, and midbrain periaqueductal gray matter/nucleus cuneiform. These nuclei influence the activity of the trigeminocervical complex and play a role in pain transmission. MD then increased again immediately following the migraine attack (66), which may reflect an increase in endogenous analgesic ability.

According to biochemical and functional imaging studies, a hypothalamic and brainstem dysfunction may also underlie sleep disorders (67) and depression (68, 69). Beyond controlling alertness and awareness, the concentration of monoamines in synaptic gaps is reduced in the depressive state (70). Serotonin, which mediates several types of human behavior including sleep, mood (depression and anxiety), appetite, sexual function, and pain, may be playing a cross-cutting function (71). Similarly, the hypothalamic–pituitary–adrenal axis, which is required for appropriate sleep regulation, is also involved in depression (69).

With regard to the autonomic nervous system (ANS), multiple afferents (i.e., lateral hypothalamus, stria terminalis, periaqueductal gray, paraventricular hypothalamic nucleus and piriform cortex) carry parasympathetic signals to the superior salivatory nucleus, which can in turn activate postganglionic parasympathetic neurons in the sphenopalatine ganglion, meningeal nociceptors and the trigemino-vascular pathway (38). An increased parasympathetic tone due to altered physiological or emotional homeostasis may activate these nociceptive pathways (38, 72). Therefore, migraine is more than an isolated event of head pain and should be seen as a bio-behavioral response to restore a disturbed brain homeostasis (73) in genetically predisposed individuals (74). We believe the ANS plays a major role in migraine pathophysiology, by modulating the internal homeostasis and using an existing defensive neuronal pathway, the trigeminovascular system, to alert the individual through pain when there is an imbalance. Regarding this aspect, some patients experience more migraine attacks on weekends (75, 76), possibly due to a sudden shift in the balance between sympathetic and parasympathetic tone and their ability to adapt to a more relaxed state.

The glymphatic system, a network of perivascular channels that helps clear waste products and toxic solutes from the brain, primarily during sleep (77), may play a key role in the pathogenesis of migraine. Even though the exact mechanisms underlying this relationship remains to be fully elucidated, it should be noted that migraine patients present a poor sleep endophenotype (i.e., poor sleep quality, shorter self-reported sleep duration, poorer sleep health score, a modestly lower percentage of rapid eye movement sleep) (78, 79). Poor sleep can produce an impairment in the glymphatic flow which, in turn, leads to the accumulation of neuroexcitatory and pro-inflammatory chemicals involved in the development of migraine (80, 81). Beyond sleep deprivation, preclinical studies have reported that body posture, stress, and adrenergic tone may also modulate the activity of the glymphatic system (82, 83).

In our theoretical model, migraine results from the combined effect of unrestful sleep, sympathetic/parasympathetic imbalance with parasympathetic prevalence and an unstable or depressive mood, all of which contribute to a state of low mental energy, potentially through the exhaustion of hypothalamic homeostatic networks. If these biological factors are strictly monitored, their prompt recognition and correction may avoid migraine progression into the painful phase. Probably, the faster the correction, the lower the probability of developing migraine attacks.

In order to demonstrate this theoretical model, wearable devices may be used to monitor heart rate variability, which provides information on ANS and the balance between sympathetic and parasympathetic activity (84). Similarly, they may be useful in monitoring sleep quality and quantity (85), coupled with self-administered questionnaires, like the Pittsburgh Sleep Quality Index (86), which has already been used to assess poor sleep quality in migraine patients (59, 79). Patients should also undergo repeated polysomnographies for comprehensive assessment of sleep and evaluation of sleep disorders (87, 88). This diagnostic tool has already been used to evaluate the possible association between migraine, bruxism, sleep apnoea and other sleep disorders (88). Monitoring of pupillary light reflex and plasma catecholamines at rest and during calibrated challenges may be used as proxies of ANS balance (89). Self-administered questionnaires, for example the Beck’s Depression Inventory II (90), or the Patient Health Questionnaire-9 (91), could be employed to evaluate mood disorders.

Regular monitoring of ANS function, mood, and sleep would allow migraine patients to identify when they are nearing or already inside the high-risk area for migraine attacks.

## Conclusion

Our theoretical model, based on repeated clinical and instrumental observations, remains at the moment a pure hypothesis, which requires validation through ongoing evidence that will be the subject of upcoming research. In the near future, the first step is to confirm the existence of the high-risk area, the second step is to identify non-pharmacological strategies in order to avoid this condition, or eventually, recuperate a well state of being, through reversing the underlying pathophysiological dysfunction. Thus, preventing migraine attacks, and enhancing the understanding of their complex pathophysiology.

## Data Availability

The original contributions presented in the study are included in the article/supplementary material, further inquiries can be directed to the corresponding author.
